# The impact of foremen’s paternalistic leadership on construction workers’ safety behaviors: considering the effects of team safety climate, workers’ psychological safety and power distance

**DOI:** 10.3389/fpubh.2025.1633467

**Published:** 2025-07-24

**Authors:** Lixia Wang, Xun Luo, Hujun Li, Huihua Chen

**Affiliations:** ^1^School of Business Administration, Henan Polytechnic University, Jiaozuo, China; ^2^School of Civil Engineering, Henan Polytechnic University, Jiaozuo, China; ^3^School of Civil Engineering, Central South University, Changsha, China

**Keywords:** foremen paternalistic leadership, workers’ safety behaviors, team safety climate, workers’ psychological safety, power distance, construction workers

## Abstract

This study investigates how foremen’s paternalistic leadership (FPL) influences construction workers’ safety behaviors (WSB) in China, and how team-level and individual-level psychological mechanisms shape this relationship. Drawing on leader–member exchange theory, psychological safety theory, and cultural congruence perspectives, this research proposes a dual-mediation model involving team safety climate (TSC) and workers’ psychological safety (WPS), with power distance (PD) as a moderating variable. A cross-sectional survey was conducted with 263 frontline workers across three Chinese cities. Structural equation modeling revealed that FPL significantly enhanced WSB both directly (*β* = 0.104, *p* < 0.001) and indirectly through TSC (indirect effect = 0.195) and WPS (indirect effect = 0.204). Multiple regression analysis showed that PD moderated the impact of FPL on both mediators, with stronger associations observed among high-PD individuals. These findings contribute to safety leadership research by contextualizing paternalistic leadership within construction settings. The results also highlight culturally contingent pathways through which leadership affects safety outcomes, offering theoretical insights and practical implications for improving construction site management and training.

## 1 Introduction

In recent years, infrastructure projects such as railways, highways, and subways in China have steadily progressed according to national development plans. However, large scales of safety accidents occur during construction of these projects, because the construction operation often takes place in a dynamic and complex environment involving both human and natural factors (1), which cause significant property losses and casualties to society, the nation, and enterprises (2). For example, 490 safety incidents occurred and 531 people died in the Chinese housing and urban–rural construction sector in 2024 (3).

Extensive research has identified unsafe worker behaviors as the leading cause of construction accidents (4, 5). Accordingly, understanding the psychological and organizational drivers of workers’ safety behaviors (WSB) is a priority in construction safety management. WSB encompasses compliance with mandatory safety rules and voluntary actions that reduce accident risk (6). Prior studies have examined various antecedents of WSB across multiple levels—individual, team, organizational, and societal (7). These include factors such as safety competence (8), safety attitudes (9), safety climate (10), and family–work interface (11).

Construction sites are characterized by close, daily interactions between workers and foremen, within a highly structured and hierarchical context. Consequently, the leadership style of foremen plays a crucial role in shaping safety-related behaviors (12–15). Previous studies often focused on the prediction ability of different western-originated safety leadership styles on WSB, such as transaction leadership (16–19), transformational leadership (20–23), laissez-faire leadership (24), rule-oriented leadership (24), participative leadership (24) and temporal leadership (25). However, these frameworks may not fully account for culturally embedded leadership dynamics in non-Western contexts (26). In China, paternalistic leadership (PL)—which combines authoritarianism, benevolence, and morality—remains prevalent in construction settings (27–30). Rooted in Confucian traditions and hierarchical social norms, PL may influence workers differently than Western leadership models (30). Yet, empirical research on its effects on safety behaviors remains limited and theoretically underdeveloped.

Moreover, mechanisms such as team safety climate (TSC) and workers’ psychological safety (WPS) have been recognized as key pathways through which leadership influences worker behavior (6, 31–33). Similarly, power distance (PD)—the degree to which hierarchical authority is accepted in a culture (34)—can shape how workers perceive and respond to leadership behavior (35, 36). However, few studies have examined how these elements interact within a unified model, particularly in the Chinese construction context, where PL is dominant and cultural expectations around authority are distinct (37, 38). This presents a critical research gap: How does PL influence WSB through psychological mechanisms, and how is this process shaped by cultural values like power distance?

In response to this gap, the present study develops and tests a contextualized model linking foremen’s paternalistic leadership to construction workers’ safety behaviors. Drawing on multiple theoretical perspectives—including leader–member exchange (LMX) theory, psychological safety theory, and cultural contingency theory— this research examines the mediating roles of TSC and WPS and the moderating effect of PD. By focusing on China’s construction sector, this study offers new insights into how culturally rooted leadership styles shape safety outcomes in high-risk, hierarchical work environments. It extends existing safety literature by moving beyond Western-centric leadership models and integrating psychological and cultural variables into a unified explanatory framework (39, 40).

## 2 Literature review and research hypotheses

### 2.1 Foremen’s paternalistic leadership and workers’ safety behaviors

Paternalistic leadership (PL) is a culturally rooted leadership style characterized by the combination of authoritarianism, benevolence, and moral integrity (29, 41). Chinese construction sites, where hierarchical relationships are often embedded in Confucian values, foremen typically adopt PL behaviors to manage their crews (42). This leadership style establishes a familial and hierarchical dynamic in which the leader exercises authority while also demonstrating personal concern and moral example.

From a theoretical perspective, PL affects workers’ behaviors not only through direct authority but also via relational and psychological mechanisms. According to leader–member exchange (LMX) theory, high-quality leader–subordinate relationships foster trust, obligation, and mutual respect (43, 44), which in turn promote discretionary behaviors such as voluntary safety compliance. Moreover, under social exchange theory, leaders who display care and fairness motivate workers to reciprocate with positive organizational behaviors, including compliance with safety norms (45, 46).

In the context of construction safety, where formal rules alone may be insufficient to guide behavior on dynamic worksites, the relational dimensions of PL—especially benevolence and moral leadership—may inspire a stronger internalization of safety values. Workers are likely to engage in proactive safety behaviors not merely to avoid punishment but because of perceived loyalty and moral obligation to their leader (47, 48). Therefore, the follow-up hypothesis can be drawn:

*H*1: Foremen’s paternalistic leadership is positively associated with workers’ safety behavior.

### 2.2 Mediating effect of team safety climate

Team safety climate (TSC) refers to workers’ shared perceptions about how safety is valued, prioritized, and supported within their immediate work group (49–52). In construction teams, these perceptions are primarily shaped by the observable behaviors and communication patterns of frontline supervisors (53–56). When foremen regularly stress safety, respond seriously to unsafe acts, and actively model compliance, these phenomena convey consistent cues that safety is a core team priority (13).

According to social information processing theory, individuals develop behavioral expectations and perceptions based on the social cues received from their environment (57). In construction work, which is dynamic, unpredictable, and often lacking detailed standard procedures, these signals become even more important. Workers typically observe how their foreman handles unsafe behavior, enforces rules, or responds to near-miss incidents to form a sense of how seriously safety is treated (58, 59). When these leader behaviors are consistent and supportive of safety, workers absorb this information and build a collective sense that safety is not only valued but expected (10). Paternalistic leaders, by blending authority with benevolence, play a unique role in this signaling process (30). Their concern for workers’ well-being lends credibility to safety expectations, while their moral integrity adds legitimacy to enforcement. Over time, such behaviors create a shared understanding of safety within the team. In this way, paternalistic leadership helps generate a normative climate that reinforces safety as a shared team value.

According to prior studies on safety climate (10, 60), a strong team safety climate increases employees’ awareness of occupational risks, encourages adherence to safety guidelines, and ultimately lowers the risk of workplace incidents (4, 61, 62). Additionally, an encouraging safety environment enhances job satisfaction, which in turn motivates employees to take a more active role and engage more willingly in safety initiatives (21, 63, 64), thereby improving work efficiency and enhancing overall safety performance. Hence, team safety climate positively affects workers’ safety behavior. Thus, according to the previous argument, this study presumes the following hypotheses:

*H*2: Foremen’s paternalistic leadership is positively associated with team safety climate.

*H*3: Team safety climate is positively associated with workers’ safety behavior.

*H*4: Team safety climate mediates the linkage between foremen’s paternalistic leadership and workers’ safety behavior.

### 2.3 Mediating effect of workers’ psychological safety

Psychological safety reflects a worker’s belief that team members can express concerns, admit mistakes, and ask questions without fear of embarrassment or punishment (65, 66). On construction sites—where risks are high, mistakes can be costly, and authority structures are pronounced—this sense of interpersonal safety is essential for promoting voluntary safety behaviors such as reporting hazards, intervening in unsafe acts, and voicing suggestions (67–69).

According to psychological safety theory, interpersonal risk-taking is a function of the perceived social cost of speaking up or acting differently in a group (70). In construction teams, workers may hesitate to report problems or challenge unsafe behavior due to fear of negative consequences, especially when foremen are seen as punitive or indifferent. Paternalistic leadership helps mitigate this risk by providing a consistent sense of emotional protection. When leaders show genuine interest in workers’ well-being, handle mistakes with understanding, and demonstrate moral consistency, workers feel respected as individuals (67, 71). This perception reduces the psychological barriers to communication and engagement (72).

Research in innovation management suggests that individuals are more inclined to act autonomously and make decisions based on personal judgment in environments where psychological safety is high (73–75). Thus, construction workers are less influenced by external pressures and more inclined to engage in safe behaviors when valuing safety. Additionally, a psychologically safe atmosphere promotes mutual trust among team members, which facilitates the exchange of safety-related knowledge and skills (76–78), and safety knowledge and skills serve as fundamental prerequisites for engaging in safe behaviors. Therefore, based on the aforementioned analyses, the following hypotheses can be drawn.

*H*5: Foremen’s paternalistic leadership is positively associated with workers’ psychological safety.

*H*6: Workers’ psychological safety is positively associated with their safety behavior.

*H*7: Workers’ psychological safety mediates the relationship between paternalistic leadership and workers’ safety behavior.

### 2.4 The moderating effect of power distance

Power distance (PD) refers to the extent to which individuals accept and expect power inequalities within social or organizational contexts (34, 79). High power distance environments typically feature strict hierarchies and limited subordinate autonomy, while low power distance cultures favor participatory leadership and egalitarian relationships (80). Meta-analytical evidence also highlights its role in shaping leadership outcomes, particularly in domains where obedience to authority directly affects rule compliance, such as occupational safety (39). Given that paternalistic leadership combines authority with benevolence (28), PD may critically shape how paternalistic leadership translates into team safety climate and psychological safety.

The effectiveness of paternalistic leadership may depend on how well its hierarchical nature aligns with the follower’s PD orientation. According to cultural congruence theory, leadership behaviors are more likely to be accepted and internalized when leaders are consistent with the subordinate’s cultural values and expectations (81). Workers high in PD are more likely to perceive paternalistic leadership as appropriate and legitimate, whereas low PD workers may interpret such behavior as over controlling or intrusive (82).

This alignment influences how workers interpret leadership signals and form collective perceptions. In the case of team safety climate, social information processing theory suggests that individual uses social cues—especially those from supervisors—to construct shared beliefs about organizational priorities (55, 83). When PD is high, workers are more attentive to leadership cues and less likely to question authority, thereby amplifying the effect of paternalistic leadership on the formation of a strong safety climate. In contrast, low PD workers may discount or reinterpret the same signals, weakening the emergence of shared norms around safety.

Similarly, in the development of workers’ psychological safety, power distance shapes how workers perceive interpersonal risks. Psychological safety theory emphasizes that individuals assess whether it is safe to speak up based on interpersonal trust and anticipated leader response (70). High PD workers are more likely to defer to leadership judgments and view benevolent authority as protective rather than punitive (29, 30). This fosters a stronger sense of psychological safety under paternalistic leadership. In contrast, low PD workers may expect autonomy and mutual dialog; if leaders exercise top-down control—even with good intentions—team members may feel constrained, thereby weakening the safety signal.

Therefore, according to the above analyses, this research proposes the following hypotheses:

*H*8: Power distance can positively moderate the linkage between foremen’s paternalistic leadership and team safety climate.

*H*9: Power distance can also positively moderate the linkage between foremen’s paternalistic leadership and workers’ psychological safety.

**Figure 1:**
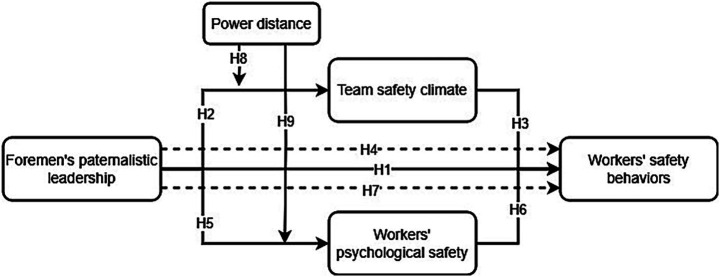
The conceptual model of this research.

In consequence, according to the aforementioned hypotheses, a conceptual model is developed for this research, which was presented in Figure 1.

## 3 Research methodology

### 3.1 Survey procedure and participants

This study focused on construction workers as participants and utilized a self-administered survey to gather data. The research instrument was divided into two parts. Part 1 included multiple-choice items designed to collect demographic information, such as gender, age, educational level, and year of work experience. Part 2 featured measurement scales assessing previously-highlighted variables, including FPL, TSC, WPS, PD, and WSB.

Fellows and Liu (84) argued that construction workers are often hard to access, and convenience sampling was frequently selected as the main sampling technique. To ensure effective access to the target population, data collection was carried out in three Chinese cities: Jiaozuo, Zhengzhou, and Changsha. The reasons why this research selected these three cities are that the research group can only access to workers in these cities because of the previous cooperation with some construction enterprises in these cities. This survey strategy helps us to gain a higher response rate and more reliable data. An online platform, Wenjuanxing, was chosen to host the digital version of the survey because many previous studies selected it as an online survey platform (25, 85). Initially, the questionnaire was sent to project managers who had collaborated with the research team. These individuals then forwarded the link to the workers under their supervision. To ensure respondent anonymity, surveys do not require personally identifiable information. Participants were informed that their responses were confidential and would be used solely for academic research purposes. Additionally, all surveys were completed privately by the workers without supervision from their foremen or project managers. The data collection period spanned from November 2024 to December 2024, lasting approximately 2 months. A total of 287 responses were received. After filtering out incomplete or invalid submissions, 263 usable responses were retained for further analysis. Table 1 provides an overview of the respondents’ demographic profiles.

**Table 1:** Participants’ demographic data statistics.

Variables	Options	Numbers	Percentage (%)
Gender	Male	248	94.30
	Female	15	5.70
Educational level	Primary school or below	71	27.00
	Junior high school	123	46.77
	High school or technical secondary school	56	21.29
	University or above	13	4.94
Age	≤20	4	1.52
	20–30	61	23.19
	31–40	72	27.38
	41–50	107	40.68
	>50	19	7.23
Year of work experiences	≤1	5	1.90
	2–4	41	15.59
	5–7	73	27.76
	8–10	51	19.39
	>10	93	35.36

### 3.2 Measurement instruments

Foremen’s paternalistic leadership was assessed using a scale developed by Cheng et al. (30), consisting of 12 items distributed across three dimensions: authoritarianism, benevolence, and moral virtue. This instrument has been extensively applied in organizational studies within the Chinese context. An example item reads: “My leader cares about my personal and professional well-being like a family elder.”

Team safety climate was evaluated with the scale introduced by Dedobbeleer and Beland (86), which contains nine items grouped into two factors: management commitment and worker participation. One sample item states: “Management considers safety to be a top priority.”

To measure workers’ psychological safety, the unidimensional scale proposed by Liang et al. (87) was employed. A representative statement from this scale is: “I feel safe expressing my opinions in front of team members.”

Power distance was captured using a five-item scale originally designed by Hofstede (88). As a unidimensional construct, it includes statements such as: “We should follow our superiors’ instructions without asking questions.”

Workers’ safety behavior was evaluated using the scale developed by Neal and Griffin (89), comprising six items across two dimensions: safety compliance and safety participation. A typical example is: “I comply with all safety rules at work.”

All aforementioned concepts were rated on a five-point Likert-type scale, ranging from 1 (strongly disagree) to 5 (strongly agree).

Several variables were selected as control variables because of their latent effects on WSB. These variables consist of workers’ gender (1 = male, 2 = female), educational level (1 = primary school or below; 2 = junior high school, 3 = senior high school or technical secondary school, 4 = university or above), and year of work experience (1 for ≤1, 2 for 2–4, 3 for 5–7, 4 for 8–10, 5 for >10).

### 3.3 Data analysis methods

To analyze the collected data, a range of statistical techniques was applied, including confirmatory factor analysis (CFA), structural equation modeling (SEM), and multiple regression analysis (MRA). CFA was conducted to assess the construct validity of the measurement model (90). SEM was utilized to evaluate both direct and mediating relationships (91). Specifically, an initial SEM model (SEM1) was developed to examine the impact of FPL on WSB. A second SEM model (SEM2), incorporating Bootstrap resampling techniques, was then constructed to investigate the mediating effects of TSC and WPS.

MRA was a widely-accepted statistical technique to test the moderating mechanism among variables (92, 93), thus this research performed MRA to explore the moderating effects of PD. Compared to the moderation analyses by incorporating an established latent interaction term in SEM, the moderation analysis in MRA is more convenient and easily conducted (94). Besides, the statistical approach to comprehensively test main effect, mediating effect and moderation effect by mixing SEM and MRA is well established in previous research (25, 95). Two baseline models (MRA1 and MRA2) were first established to assess the main effects among FPL, TSC, WPS, and PD. Subsequently, two extended models (MRA3 and MRA4), which incorporated interaction terms, were developed to test the moderating effect of PD on these relationships.

## 4 Research results

### 4.1 Reliability and validity analysis

The collected data were imported into SPSS 23 for analysis, and Cronbach’s alpha coefficient was calculated to evaluate internal consistency. The results revealed alpha values of 0.92 for FPL, 0.93 for TSC, 0.91 for WPS, 0.89 for PD, and 0.94 for WSB—all surpassing the recommended threshold of 0.80. This suggests that the measurement instruments demonstrated strong reliability.

To examine the suitability of the data for factor analysis, the Kaiser-Meyer-Olkin (KMO) measure and Bartlett’s test of sphericity were conducted. KMO values for all variables exceeded 0.70, and Bartlett’s test yielded statistically significant results (*p* < 0.05), indicating that the dataset met the assumptions required for further exploratory and confirmatory analyses.

CFA was carried out using AMOS 23 to assess the construct validity of the measurement scales. Six measurement models (including the hypothesized model and 5 alternative models) were established for CFA, and the calculated results were presented in Table 2. As presented, our hypothesized model demonstrates a better fit (χ^2^/df = 2.21, GFI = 0.906, CFI = 0.929, RMSEA = 0.055). Convergent validity was evaluated through factor loadings, composite reliability (CR), and average variance extracted (AVE) based on the hypothesized model results. The calculating results are presented in Table 3. The measurement tools for FPL, TSC, and WPS exhibited acceptable levels of convergent validity. However, two items related to WSB were eliminated due to factor loadings below 0.70. The revised four-item version of the WSB scale subsequently passed the convergent validity criteria.

**Table 2:** Results of the CFA for the six models.

Models	χ^2^/df	GFI	CFI	TLI	RMSEA
Five-factor hypothesized model (including FPL, TSC, WPS, WSB and PD)	2.21	0.906	0.929	0.932	0.055
Four-factor model 1 (FPL and PD were collapsed into one factor)	4.31	0.779	0.844	0.837	0.082
Four-factor model 2 (TSC and PD were collapsed into one factor)	4.86	0.753	0.834	0.824	0.088
Three-factor model (FPL, TSC, and PD were collapsed into one factor)	7.45	0.714	0.752	0.722	0.114
Two-factor model (WPS and WSB were collapsed into one factor based on the three-factor model)	10.31	0.618	0.639	0.599	0.137
One-factor model	11.295	0.616	0.599	0.556	0.144

**Table 3:** Analysis of the CV of the measurement scale.

Variables/dimensions		Measurement item	Factor loading	Reliability	CR	AVE
Foremen’s paternalistic leadership	Authoritarianism	AQ1	0.791	0.82	0.858	0.602
		AQ2	0.773	0.78		
		AQ3	0.815	0.81		
		AQ4	0.723	0.73		
	Benevolence	BQ1	0.827	0.81	0.877	0.642
		BQ2	0.796	0.8		
		BQ3	0.764	0.77		
		BQ4	0.816	0.82		
	Morality	MQ1	0.785	0.79	0.872	0.630
		MQ2	0.813	0.82		
		MQ3	0.797	0.81		
		MQ4	0.778	0.78		
Team safety climate	Management commitment	MCQ1	0.851	0.84	0.923	0.706
		MCQ2	0.843	0.83		
		MCQ3	0.826	0.81		
		MCQ4	0.817	0.81		
		MCQ5	0.863	0.79		
	Workers’ involvement	WPQ1	0.824	0.81	0.880	0.648
		WPQ2	0.817	0.8		
		WPQ3	0.795	0.78		
		WPQ4	0.783	0.77		
Workers’ psychological safety		PSQ1	0.831	0.82	0.905	0.657
		PSQ2	0.817	0.8		
		PSQ3	0.786	0.79		
		PSQ4	0.793	0.8		
		PSQ5	0.824	0.83		
Power distance		PDQ1	0.811	0.81	0.901	0.644
		PDQ2	0.832	0.79		
		PDQ3	0.792	0.83		
		PDQ4	0.784	0.87		
		PDQ5	0.793	0.85		
Workers’ safety behavior		SBQ1	0.883	0.87	0.913	0.723
		SBQ2	0.846	0.83		
		SBQ3	0.814	0.81		
		SBQ4	0.857	0.84		

AQ1–AQ4 are items measuring authoritarianism; BQ1–BQ4 measure benevolence; MQ1–MQ4 measure morality; MCQ1–MCQ5 measure management commitment; WPQ1–WPQ4 measure worker participation; PSQ1–PSQ5 measure WPS; PDQ1-PDQ5 measure PD, and SBQ1–SBQ4 measure WSB.

The square roots of the AVE for each construct, along with their correlations to other variables, were computed. These findings are summarized in Table 4. As illustrated, the square roots of the AVE for all variables exceed their corresponding Pearson correlation coefficients with other variables (dimensions), confirming adequate discriminant validity among the constructs.

**Table 4:** Differential validity analysis of the measurement scale.

Variables	Authoritarianism	Benevolence	Morality	Management commitment	Workers’ involvement	Workers’ psychological safety	Power distance	Workers’ safety behaviors
Authoritarianism	0.776*							
Benevolence	0.534	0.801*						
Morality	0.527	0.613	0.794*					
Management commitment	0.313	0.243	0.173	0.840*				
Workers’ involvement	0.342	0.312	0.213	0.657	0.805*			
Workers’ psychological safety	0.217	0.311	0.314	0.213	0.134	0.812*		
Power distance	0.213	0.345	0.324	0.334	0.367	0.439	0.802*	
Workers’ safety behaviors	0.472	0.314	0.342	0.363	0.453	0.443	0.231	0.850*

*denotes they are the square roots of the AVE for each construct.

### 4.2 Common method variance

The data were also imported into SPSS 23 to test common method variance; the Harman’s one-factor test shows that the first principal factor only explains 18.43% of the total variance (< 40%), which indicates that the common method variance is not significant.

### 4.3 Test of main effects

**Figure 2:**
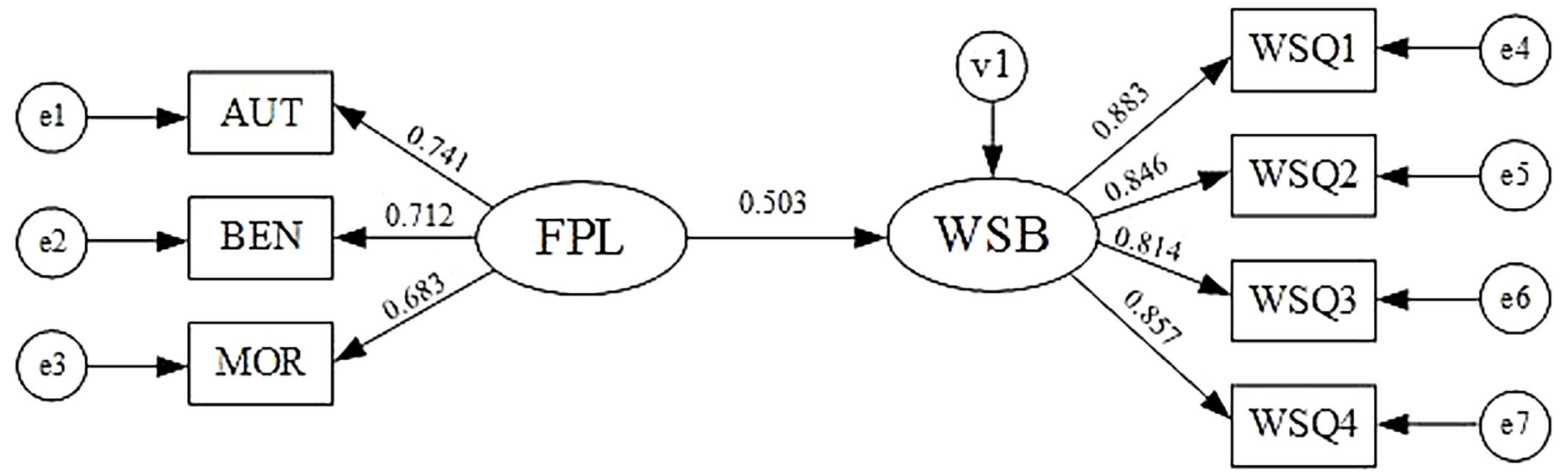
The calculated results of SEM 1. AUT refers to authoritarianism; BEN refers to benevolence; and MOR refers to morality. The values of AUT, BEN and MOR are calculated based on the item parceling technique (116).

SEM1 was established to investigate the direct impact of FPL on WSB. The model demonstrated acceptable fit based on standard fit indices: χ^2^/df = 2.35, GFI = 0.915, CFI = 0.943, and RMSEA = 0.053. These indicators collectively confirm that SEM1 achieved a satisfactory model fit. No post-hoc model modifications were made, as all proposed pathways were theoretically grounded and statistically significant. Moreover, modification indices did not indicate the need for additional paths to improve model fit. The results of SEM1 were presented in Figure 2.

Table 5 presents the path coefficients and their significance levels within SEM1. The analysis reveals that the regression coefficient for the relationship between FPL and WSB is statistically significant at *p* < 0.001, suggesting a strong positive association. Consequently, Hypothesis H1 is supported.

**Table 5:** Path regression coefficient and significance of SEM1.

Model	Path	Path coefficient	S. E.	C. R.	*p*
SEM1	FPL → WSB	0.503	0.043	11.47	***

“***” indicates that the value is less than 0.001.

### 4.4 Test of mediating effects of team safety climate and workers’ psychological safety

**Figure 3:**
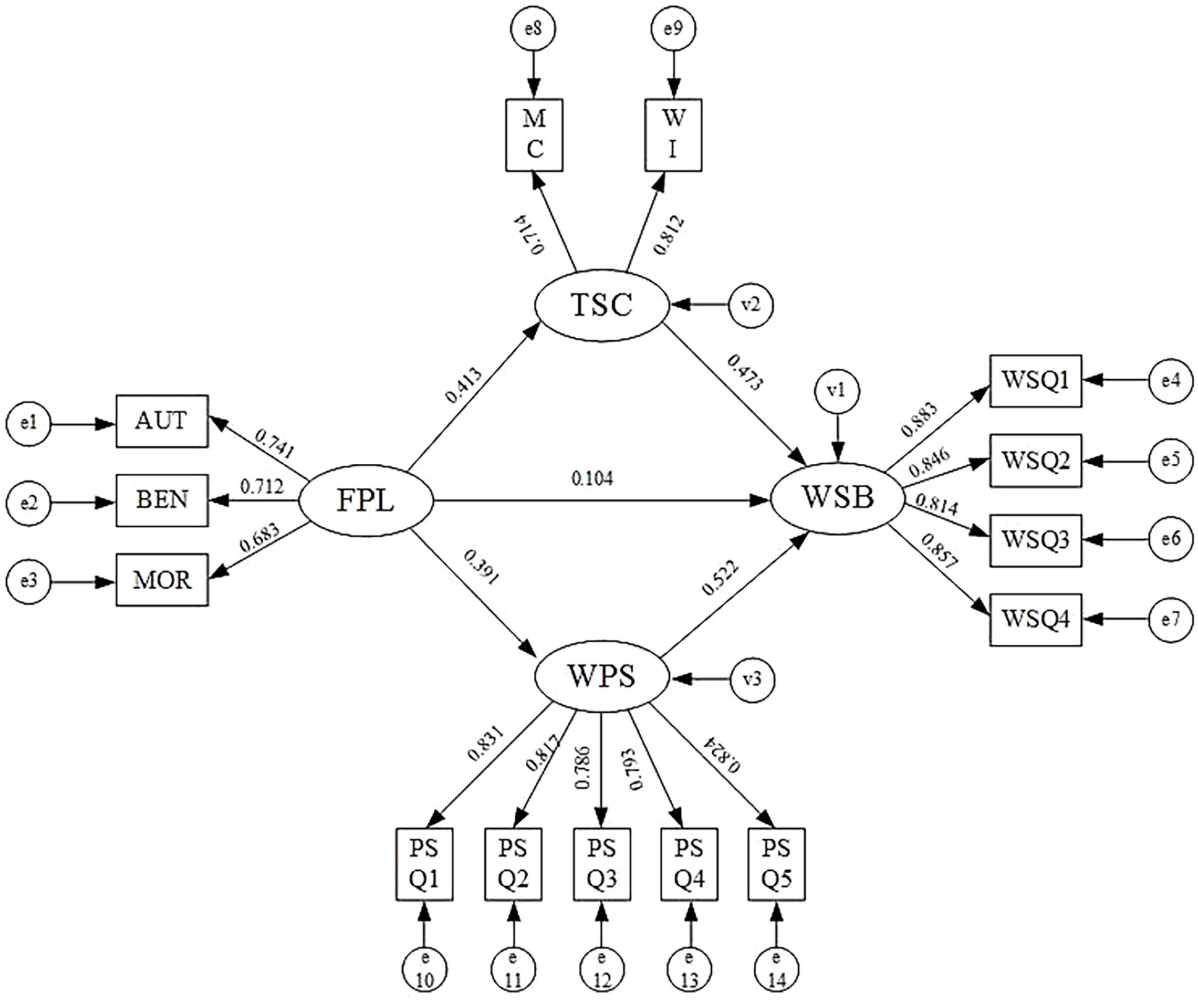
The calculated results of SEM 2. AUT refers to authoritarianism; BEN refers to benevolence; MOR refers to morality, MC refers to management commitment; and WI refers to workers’ involvement. The values of AUT, BEN, MOR, MC and WI are calculated based on the item parceling technique (116).

To explore the mediating effects of TSC and WPS on the relationship between FPL and WSB, a parallel mediation structural equation model (SEM2) was developed in this research. The model exhibited an acceptable fit: χ^2^/df = 2.18 < 3, GFI = 0.912 > 0.9, CFI = 0.931 > 0.9, and RMSEA = 0.052 < 0.08. Table 6 displays the path coefficients and their significance levels, while Table 7 summarizes the estimates and statistical significance of the indirect effects. The results of SEM2 were presented in Figure 3.

**Table 6:** Significance analysis of SEM2 regression coefficient.

Models	Path	Path coefficient	S. E.	C. R.	Bootstrap 5,000				*p*
					Bias-corrected		Percentile		
					Lower	Upper	Lower	Upper	
SEM 2	FPL → TSC	0.413	0.035	10.83	0.287	0.524	0.303	0.509	***
	TSC → WSB	0.473	0.033	13.65	0.347	0.617	0.356	0.607	***
	FPL → WPS	0.391	0.041	9.53	0.279	0.501	0.286	0.487	***
	WPS → WSB	0.522	0.043	15.67	0.378	0.679	0.394	0.657	***
	FPL → WSB	0.104	0.042	4.21	0.078	0.139	0.082	0.131	***

“***” indicates that the value is less than 0.001.

**Table 7:** Significance of SEM2 mediating effect.

Effect type	Path coefficient	S. E.	C. R.	Bootstrap 5,000				*p*
				Bias-corrected		Percentile		
				Lower	Upper	Lower	Upper	
FPL → TSC → WSB	0.195	0.047	4.16	0.143	0.258	0.149	0.251	***
FPL → WPS → WSB	0.204	0.053	4.01	0.147	0.264	0.153	0.259	***
Total indirect effect	0.399	0.048	8.39	0.274	0.519	0.303	0.511	***
Direct effect	0.104	0.042	4.21	0.078	0.139	0.082	0.131	***
Total effect	0.503	0.043	11.47	0.355	0.658	0.387	0.645	***

“***” indicates that the value is less than 0.001.

After 5,000 bootstrap resamples, the regression coefficients for the paths from PL to TSC (0.413) and from TSC to WSB (0.473) were both highly significant (*p* < 0.001). These findings confirm that FPL enhances TSC, which in turn promotes safer behaviors among workers. As a result, Hypotheses H2 and H3 are validated. Moreover, these results suggest that TSC partially mediates the link between FPL and WSB, supporting Hypothesis H4. The indirect effect through this pathway is estimated at 0.195, indicating a meaningful impact.

In addition, after 5,000 bootstrap samples, the regression coefficients for the paths from FPL to WPS and from WPS to WSB were 0.391 and 0.522, respectively, both statistically significant (*p* < 0.001). This indicates that FPL contributes to improving WPS, which subsequently leads to enhanced WSB. Thus, Hypotheses H5 and H6 are supported. Consequently, WPS acts as a mediator in the relationship between FPL and WSB, confirming Hypothesis H7. The indirect effect via this mechanism is calculated as 0.204.

Although the indirect effect sizes (0.195 and 0.204) appear moderate, these paths represent meaningful improvements in workers’ safety behavior, particularly in settings where small behavioral shifts can translate into significant reductions in accident risk (6). These effect sizes are comparable to or exceed those reported in prior safety behavior research using similar constructs (11, 25, 95). Besides, the direct effect of FPL on WSB in SEM2 was 0.104 and remained statistically significant, suggesting that FPL also exerts a direct positive impact on WSB. A comparison of the three pathways reveals that the direct impact of FPL on WSB is smaller than its indirect effects, implying that the overall impact of leadership on safety behavior primarily operates through these two psychological mechanisms.

### 4.5 Test of the moderating effect of power distance

To examine the moderating role of PD in the relationship between FPL and TSC, two multiple regression models (MRA 1 and MRA 2) were established. In MRA 1, FPL and PD served as independent variables, with TSC as the outcome variable. Building on this, MRA 2 introduced an interaction term (i.e., FPL × PD) to assess the moderating influence.

The results from both models are presented in Table 8. A statistically significant change was found in *F* value (ΔF = 18.753), suggesting that MRA 2 provided a better fit, as reflected by an increase in the adjusted R^2^ by 0.036. Notably, the coefficient for the interaction term was 0.124 and reached statistical significance, indicating that PD positively moderates the effect of FPL on TSC.

**Table 8:** Moderating effect of PD on the pathway of FPL influencing TSC.

Models	Variables	Beta	*t*	Sig.	*R^2^*	Adjusted *R^2^*	Δ*R^2^*	Δ*F*	Sig.
MRA 1	FPL	0.421	8.432	0.000	0.413	0.403		103.325	0.000
	PD	0.338	7.365	0.000					
MRA 2	FPL	0.447	6.532	0.000	0.449	0.436	0.036	18.753	0.000
	PD	0.329	7.721	0.000					
	FPL × PD	0.124	3.937	0.000					

Furthermore, MRA 3 and MRA 4 were developed to investigate the moderating effect of PD on the linkage between FPL and WPS. In MRA 3, FPL and PD were treated as independent variables, with WPS serving as the dependent variable. MRA 4 extended this model by incorporating an interaction term (i.e., FPL × PD) to assess the moderating effect.

The outcomes of these two models are summarized in Table 9. The change in *F* value (ΔF = 16.929) was found to be statistically significant, indicating that MRA 4 offered a better model fit, as evidenced by an increase of 0.041 in the adjusted R^2^. Additionally, the coefficient for the interaction term was 0.175, which was also statistically significant. This result suggests that PD positively moderates the relationship between FPL and WPS.

**Table 9:** Moderating effect of PD on the pathway of PL influencing WPS.

Models	Variables	Beta	*t*	Sig.	*R^2^*	Adjusted *R^2^*	Δ*R^2^*	Δ*F*	Sig.
MRA 3	FPL	0.387	8.016	0.000	0.356	0.347		107.231	0.000
	PD	0.427	6.636	0.000					
MRA 4	FPL	0.418	7.863	0.000	0.397	0.383	0.041	16.929	0.000
	PD	0.431	5.992	0.000					
	FPL × PD	0.175	3.351	0.000					

## 5 Discussion and managerial implications

### 5.1 Discussion

#### 5.1.1 Direct effect of foremen’s paternalistic leadership on workers’ safety behavior

This study identifies a robust direct effect of foremen’s paternalistic leadership (FPL) on construction workers’ safety behavior (WSB), contributing to ongoing debates on the leadership–safety link. Prior research focused heavily on Western-originated leadership types as key antecedents of safety behavior (26)—highlighting mechanisms like inspirational motivation, contingent reinforcement, and explicit communication of safety expectations (67, 96–98). These models, frequently studied in Western contexts, treat safety compliance and participation as outcomes of rational alignment with organizational goals or charismatic influence. However, our findings suggest that in the Chinese construction context—where informal hierarchical relationships and Confucian values are deeply rooted—FPL may be equally or more effective. Unlike transformational leadership, which relies on abstract vision and individualized consideration, PL integrates emotional closeness with moral discipline and paternal guidance, aligning better with local cultural expectations (28, 30). Unlike transactional leadership, which depends on explicit rewards or punishments, PL fosters safety through invoking loyalty, obligation, and moral responsibility.

While both transformational and paternalistic leadership aim to inspire in-role and extra-role behaviors, the mechanisms differ: Western-originated leadership theories tend to emphasize autonomy and internalized motivation (67, 99), whereas FPL leverages relational bonds and role-based moral obligation. This distinction underlines the importance of contextualizing leadership effectiveness within culturally embedded expectations. This divergence from mainstream leadership models underscores the importance of contextualizing leadership effects. In particular, the results reinforce Leader–Member Exchange (LMX) theory (44) as a compelling framework: FPL cultivates high-quality relationships—characterized by trust, respect, and socio-emotional reciprocity—which motivate workers to enact discretionary safety behaviors. Recent empirical studies support this relational mechanism. For example, Zhang et al. (48) found that benevolent and moral dimensions of PL directly increased safety participation among high-speed railway drivers in China, with LMX mediating this effect. Similarly, a study of transit bus drivers reported a positive direct impact of PL on safety performance (100). These studies extend our findings beyond construction to other high-risk, hierarchical Chinese workplaces, reinforcing the cross-context validity of PL–LMX–safety linkage. Beyond LMX, our findings also align with Conservation of Resources (COR) theory (101), suggesting that FPL provides socio-emotional resources that reduce stress and preserve mental energy for focusing on safety. Specifically, the observed direct effect of FPL on WSB (*β* = 0.104, *p* < 0.01) suggests that such leadership offers a stabilizing relational context that mitigates the cognitive load of risk perception, consistent with COR’s emphasis on resource preservation under stress (102).

#### 5.1.2 Mediating role of team safety climate

Our results confirm that team safety climate (TSC) significantly mediates the relationship between paternalistic leadership and safety behavior, aligning with recent empirical findings in Chinese construction settings (61). Notably, Song et al. (103) demonstrated that the kindness and virtue dimensions of paternalistic leadership positively influence safety behavior through safety climate in China. This complements Zohar’s (49) foundational view of TSC and shows how culturally grounded leadership shapes localized safety norms. Furthermore, Cong et al. (104) implied that LMX-mediated leadership fosters a positive safety climate by improving informal safety communication on construction sites.

This supports our argument that benevolence, moral leadership, and ethically bounded authority coalesce into powerful social cues that structure team safety perceptions. In addition, Xia et al. (10) conducted a systematic review of 178 construction safety-climate studies, confirming that supervisor behavior and communication patterns are the strongest predictors of TSC. Such findings reinforce our claim that paternalistic foremen act as key architects of climate through daily interactions and cultural resonance.

Theoretically, this process aligns with Social Information Processing (SIP) theory (57), which posits that employees derive behavioral expectations from leaders’ expressed values and actions. Our findings, especially the significant indirect effect from FPL to WSB via TSC (*β* = 0.195, *p* < 0.01), illustrate how workers interpret repeated leadership behaviors as normative signals that shape collective safety meaning. SIP theory is thus not only validated in this context, but extended by our evidence that the style of leadership (relational and moral rather than technical or transactional) enhances the salience of such social cues. Moreover, while not original to our hypothesis, Referent Cognitions Theory (105) applies here: when workers perceive leaders’ moral and consistent actions as appropriate, these workers view safety expectations as legitimate, further solidifying TSC. In particular, fairness and moral leadership satisfy workers’ referential comparisons—between what “is” and what “should be”—thereby motivating compliance and climate conformity (106). This mechanism helps explain why workers under morally upright foremen internalize safety messages more deeply, compared to contexts where managerial behavior is inconsistent or instrumental. Compared to Western findings, which often emphasize top-down rule enforcement or transformational vision as key to safety climate (50, 107), our results suggest that relational credibility and cultural legitimacy may be more influential in decentralized, peer-driven settings such as Chinese construction teams.

#### 5.1.3 Mediating role of workers’ psychological safety

This study finds that workers’ psychological safety (WPS) mediates the relationship between foremen’s paternalistic leadership (FPL) and construction workers’ safety behavior (WSB), indicating that psychological safety is particularly vital in construction environments, where tight schedules, complex tasks, and hierarchical structures often suppress interpersonal risk-taking. Existing research consistently shows that psychological safety promotes open communication, near-miss reporting, hazard identification, and peer intervention—all key components of discretionary safety behavior (67, 108). Specifically, Demirkesen et al. (109) found that Lean construction practices—through mechanisms like respect-for-person and continuous improvement—enhanced safety behavior primarily via boosting WPS. Their study in US construction contexts confirms that WPS is a necessary intermediary between process changes and safety outcomes.

This research confirms this mediating mechanism: the path coefficient from FPL to WPS was 0.391 (*p* < 0.001), and from WPS to WSB was 0.522 (*p* < 0.001), with a significant indirect effect of 0.204 based on 5,000 bootstrap samples. These findings align with psychological safety theory, which emphasizes the role of trust-based environments in enabling proactive and discretionary work behaviors (70). FPL, through its relational focus, reduces the social costs of speaking up, thereby enabling workers to engage more fully in safety-oriented actions. Our results extend the application beyond traditional domains like healthcare or high-risk teams.

Moreover, prior research on LMX offers conceptual support for the psychological processes underlying the FPL–WPS–WSB pathway. High-quality LMX relationships are characterized by mutual trust, emotional support, and respect—conditions shown to enhance psychological safety (14, 110). Since paternalistic leadership emphasizes benevolence and moral obligation, it likely fosters a similar relational atmosphere, which makes workers more confident that their safety-related concerns will be accepted rather than punished. These insights offer an explanatory basis for why FPL enhances WPS, even in rigid or hierarchical site environments.

Our findings also resonate with Conservation of Resources (COR) theory (101, 111). Psychological safety can be seen as a personal resource that reduces emotional exhaustion and anxiety, allowing workers to allocate more attention and energy toward safety tasks. FPL, by creating a psychologically safe environment, helps workers preserve their internal resources, mitigating stress and increasing cognitive bandwidth for hazard monitoring and safe decision-making.

#### 5.1.4 Moderating role of power distance

This study finds that power distance (PD) significantly moderates the effects of foremen’s paternalistic leadership (FPL) on both team safety climate (TSC) and workers’ psychological safety (WPS), offering important insights into how cultural value orientations shape leadership effectiveness in construction safety contexts. PD refers to the extent to which individuals accept hierarchical authority and unequal power distributions within organizations (34). In high-PD cultures, subordinates are more likely to defer to authority, accept asymmetry in leader–follower relationships, and conform to top-down directives (112).

Our findings show that FPL is more effective in enhancing both TSC and WPS when workers hold higher PD values. This supports Cultural Congruence Theory (81), which posits that leadership is more readily accepted and internalized when it aligns with followers’ cultural expectations. In high-PD contexts, the hierarchical structure implicit in PL—particularly its authoritarian component—is seen as legitimate and reasonable. Consequently, workers are more responsive to leadership signals and more likely to adopt the behavioral norms conveyed by their foremen.

These effects are further reinforced by Social Information Processing theory (57), which suggests that individuals look to salient social cues—especially from those in authority—to interpret acceptable behaviors. Our results support this logic, as the interaction terms in the regression models were significant (FPL × PD → TSC: *β* = 0.124, *p* < 0.001; FPL × PD → WPS: *β* = 0.175, *p* < 0.001), indicating that workers with higher PD orientations were more sensitive to leaders’ signals. In these settings, paternalistic cues such as moral guidance or benevolent protection are more likely to be perceived as legitimate and appropriate, thereby amplifying their impact on team and individual perceptions. Recent studies also support this mechanism: in high-PD teams, workers are more attuned to supervisory cues and less likely to reinterpret or question them, amplifying the influence of leadership on safety outcomes (113). However, in lower-PD contexts, paternalistic behaviors may be perceived as controlling or intrusive, undermining their positive effects on psychological and normative safety climates. This aligns with Guo et al. (114), who found that PD moderates the effect of inclusive leadership on employee voice—diminishing it when PD is high.

While our study conceptualizes PD primarily as an individual difference variable, it is important to note that this variable remains deeply embedded in broader cultural patterns. Existing literature (34, 112) views PD as a relatively stable cultural trait. However, emerging scholarship suggests that PD may also vary across individuals due to factors such as education, industry experience, and exposure to global norms. Although our study does not directly examine these sources of variation, future research could explore how within-group differences in PD shape the effectiveness of leadership behaviors. Such work would further refine our understanding of PD not as a fixed cultural attribute, but as a dynamic, context-sensitive moderator.

### 5.2 Management implication

The findings of this study provide actionable guidance for improving safety outcomes on construction sites through culturally grounded leadership strategies. To maximize practical relevance, this research offers three targeted recommendations while addressing potential barriers in cross-cultural settings.

First, construction firms operating in high-risk environments should move beyond generic leadership development and introduce modular training programs focused on scenario-based paternalistic leadership (PL). Instead of emphasizing only general authority or empathy, such training should simulate on-site dilemmas—e.g., responding to unsafe behavior while maintaining relational trust—to help foremen integrate moral integrity with discipline in practice. In parallel, formal performance evaluations should include 360-degree safety behavior assessments from both supervisors and workers, ensuring that PL effectiveness is reinforced through organizational incentives rather than informal approval alone (13).

Second, to cultivate a psychologically safe team climate that sustains safety behavior, site-level managers must institutionalize structured peer-review and “speak-up” routines. For example, weekly “safety listening huddles” can be co-led by foremen and peer champions to normalize open dialog on near-misses and unsafe acts. Unlike top-down safety audits, these grassroots safety conversations democratize risk perception while preserving PL’s role as a relational anchor. To make this effective, firms must also protect whistleblowers through formal “non-retaliation clauses” written into project protocols, particularly for subcontracted laborers who may be more vulnerable to retaliation (70).

Third, the moderating effect of power distance (PD) highlights the need for cultural calibration in multinational projects. While PL may align well with workers in East Asian or Middle Eastern settings, its hierarchical undertones may clash with egalitarian norms in European or North American teams. Multinational firms should adopt a dual-mode leadership strategy, enabling foremen to shift between culturally dominant PL and more participative, autonomy-supportive behaviors depending on team composition (40). This requires training not only in leadership style but also in cross-cultural adaptability—for example, through simulated intercultural role-play and real-time feedback tools. Additionally, project assignments should consider foremen–team cultural match as part of workforce deployment strategy (67).

However, barriers to implementing PL in multinational contexts must not be underestimated. Some foremen may lack moral legitimacy or interpersonal skills to enact PL credibly, especially when promoted based on technical seniority. Others may face language, trust, or role ambiguity issues when managing diverse crews unfamiliar with hierarchical relational norms. To address this, HR departments should offer cultural mentoring and language-pairing systems, linking foremen with multilingual safety liaisons who can mediate cultural misunderstandings while preserving leadership authority (115).

In sum, this study encourages firms to treat paternalistic leadership not merely as a trait of individual foremen, but as a strategically cultivable and context-sensitive management capability. When backed by institutional systems, cultural awareness, and credible support mechanisms, PL can serve as a practical lever to foster safer, more cohesive construction teams.

### 5.3 Limitations and future research

Despite this study’s theoretical and practical contributions, several limitations must be acknowledged and addressed in future research.

First, the use of a cross-sectional research design limits causal inference. Although structural equation modeling (SEM) allows examination of directional associations, it cannot fully rule out reverse causality. For example, workers with strong safety behaviors may elicit more paternalistic responses from their foremen. Future studies should consider longitudinal or experimental designs to capture the evolving relationship between leadership behaviors and safety engagement.

Second, this study relies exclusively on self-reported data collected through a single-source survey. This approach introduces potential social desirability bias, particularly in a collectivist context where workers may feel pressured to report high levels of safety compliance or favorable views of authority figures. Future research should integrate multi-source data, such as supervisor ratings, peer assessments, or behavioral safety observations, to reduce method bias and validate findings through triangulation.

Third, although this study controls for power distance (PD) as a moderator, it does not address other important individual or contextual factors, such as job stress, project complexity, or multicultural crews. Including these variables could deepen our understanding of how paternalistic leadership interacts with organizational conditions. For instance, examining whether benevolence’s safety-enhancing effect changes under time pressure versus routine work could provide a more nuanced view of leadership adaptability.

Fourth, while this study adopts an integrated view of paternalistic leadership, it does not differentiate its specific subdimensions—authoritarianism, benevolence, and moral leadership—in empirical testing. Future research should consider separating these components to explore whether benevolence enhances psychological safety while authoritarianism undermines it, especially in teams with mixed power distance values. Such research could challenge the assumption that all elements of PL function synergistically and contribute to more detailed theoretical development.

Fifth, although this study is situated in a single cultural context, future research could explore within-country variations in how paternalistic leadership operates across regions or organizational types. For instance, state-owned enterprises, private firms, and foreign-invested projects may differ in how workers respond to benevolence or authoritarian cues. Such comparative designs would help identify intra-national heterogeneity in leadership reception and cultural congruence, without assuming cross-national generalizability.

Finally, future research should move beyond confirming existing effects and consider paternalistic leadership as a dynamic, socially constructed influence. For instance, when does moral leadership become intrusive? How might generational shifts or rising individualism reshape worker expectations of foremen? At what point does care transform into control? Addressing such provocative questions would deepen our understanding of paternalism as a contested, evolving social phenomenon.

## 6 Conclusion

This study introduces and validates a conceptual model that explains how foremen’s paternalistic leadership (FPL) enhances construction workers’ safety behaviors. Grounded in Social Information Processing Theory and Psychological Safety Theory, the model identifies team safety climate and workers’ psychological safety as dual mediators, and power distance as a cultural moderator. Empirical results confirm the original research hypotheses and fulfill the stated research objectives: FPL positively influences workers’ safety behaviors, both directly and indirectly through relational and psychological mechanisms, and the strength of these effects varies with workers’ power distance orientation.

By framing paternalistic leadership as a culturally embedded leadership style—distinct from Western-originated leadership types—this study highlights its unique relevance in the hierarchical and relational context of Chinese construction teams. The findings extend existing theory by demonstrating that safety behavior is jointly shaped by shared team norms and individual psychological conditions, and by embedding leadership effects in culturally specific work dynamics. In particular, this research offers evidence that the relational (benevolence, moral virtue) aspects of FPL promote psychological safety, while authoritarianism may operate differently depending on contextual and individual factors.

Theoretically, this study contributes to cross-cultural leadership literature by unpacking how paternalistic leadership activates distinct motivational and cognitive pathways that improve safety outcomes. It also advances the integration of relational leadership theories with safety behavior research through the joint consideration of team- and individual-level mediators. Practically, the findings underscore the value of developing leadership training programs that promote respectful authority, individualized care, and moral integrity among foremen. Recognizing workers’ power distance orientation further enables managers to tailor communication and supervisory approaches, thereby fostering greater psychological engagement in safety practices.

Overall, this study reaffirms the central role of leadership in shaping construction safety performance. More importantly, it demonstrates that culturally coherent leadership models—when aligned with workers’ expectations and contextual realities—can serve as effective tools for building safer and more responsive frontline teams in high-risk industries.

## Data Availability

The original contributions presented in the study are included in the article/supplementary material, further inquiries can be directed to the corresponding author.
